# Preface to the special issue “Diverse Symbiotic Relationships between Plants and Microbes in the Phyllosphere and Rhizosphere”

**DOI:** 10.5511/plantbiotechnology.25.0000p

**Published:** 2025-09-25

**Authors:** Kei Hiruma, Akifumi Sugiyama

**Affiliations:** 1 Department of Life Sciences, Graduate School of Arts and Sciences, The University of Tokyo, 3-8-1 Komaba, Meguro-ku, Tokyo 153-8902, Japan; 2 Laboratory of Plant Gene Expression, Research Institute for Sustainable Humanosphere, Kyoto University, Uji, Kyoto 611-0011, Japan

Plants in nature interact with diverse microbes, some of which promote growth or enhance plant health by protecting against pathogens. Recently, research has progressed extensively from studying binary plant-microbe interactions to exploring complex plant-microbiome relationships. This special issue focuses on plant-microbe and plant-microbiome interactions, with an emphasis on beneficial microbes in both the phyllosphere and rhizosphere. It comprises eight review articles and ten original research articles.

The phyllosphere microbiota encompasses the diverse community of microorganisms residing on the leaf-dominated aerial surfaces of plants. This microbial ecosystem is integral to plant health, growth, and environmental interactions. Yurimoto (2025) reviewed the potential roles of C1 microorganisms that utilize two major greenhouse gases—methanol and CO_2_—in promoting plant growth. In particular, he focused on *Methylobacterium* spp., representative methanol-utilizing bacteria and dominant phyllosphere colonizers, and discussed strategies for harnessing these microbes to reduce greenhouse gas emissions and enhance plant growth. Hirata et al. (2025) reviewed bacterial adaptation to the phyllosphere environment and the mechanisms of community assembly within the phyllosphere microbiota. This review highlights bacterial functions crucial to plant growth, including phytohormone production, nutrient uptake, and pathogen resistance, with a focus on the application of these bacterial functions in field crop production. Within the diverse spectrum of phyllosphere bacteria, Grossi et al. (2025) focused on pink-pigmented facultative methylotrophs (PPFM), which include the genera *Methylobacterium* and *Methylorubrum*. These bacteria are characterized by their ability to utilize one-carbon compounds, such as methanol, as a carbon source. This review provides an overview of the plant-associated lifestyle of PPFMs, emphasizing their plant growth-promoting characteristics, such as the production and modulation of plant hormones, biological nitrogen fixation, and defense against biotic stresses. Masuda et al. (2025) conducted an analysis of the rice phyllosphere bacterial communities at three distinct growth stages—panicle initiation, heading, and harvesting—across three genotypes. By employing full-length 16S rRNA sequencing, the authors identified bacterial species and observed notable alterations in bacterial composition throughout the different growth stages. Notably, Enterobacter spp. demonstrated species-specific trends that varied across the stages.

The plant rhizosphere and root microbiota consist of microbes originating from both the surrounding soil and the plant’s associated soil environment. These microbes receive photosynthetically derived compounds from the host and, in return, confer benefits such as promoting plant growth and protecting the host from pathogens. The rhizosphere is a dynamic zone where a wide range of chemicals are exchanged between plants and microbes, shaping nutrient availability, microbial community structure, and plant defense mechanisms. Murata (2025) reviewed the diversity of plant- and microbe-derived volatiles, their underlying molecular mechanisms—including ecological aspects—and their interactions with non-volatile root exudates. Alkaloids represent one of the largest classes of plant specialized metabolites, characterized by diverse chemical structures and activities. Shimasaki and Nakano (2025) reviewed the roles of plant-derived alkaloids and other metabolites in plant-microbe (microbiome) interactions within the rhizosphere, with an emphasis on their contributions to plant fitness. Triterpenoid saponins are widely distributed metabolites in angiosperms and exhibit diverse biological and pharmacological activities. Nakamura and Sugiyama (2025) investigated saponin accumulation patterns during the development of the shade-tolerant shrub *Ardisia crenata* and reported their effects on the rhizosphere microbiome. Hemelda and Noutoshi (2025) examined the functions of root-exuded sugars as both carbon sources and signaling molecules in rhizosphere microbiome assembly, highlighting the need for accurate quantification of sugars that are rapidly metabolized by microbes and the genetic components underlying rhizosphere metabolic interactions.

Some microbes promote plant growth by enhancing host nutrient acquisition through various mechanisms. Arbuscular mycorrhizal fungi (AMF) colonize the roots of most plants and enhance growth by transferring macronutrients such as phosphorus and nitrogen via their hyphae. AMF are classified into two morphotypes—the Arum type and the Paris type. Higashi et al. (2025) investigated the relationship between AMF morphotypes and species identity using two plant hosts, tomato (*Solanum lycopersicum*) and *Lotus japonicus*. By assessing plant growth and gene expression profiles, they found that AMF species identity, rather than morphotype, determines the degree of AMF-mediated plant growth promotion and protection. Plant roots can host diverse AMF groups, such as Glomeromycotina (G-AMF) and Mucoromycotina (M-AMF), which are difficult to distinguish within root tissues. Erdenetugs et al. (2025) developed a method to isolate single vesicles from roots and sequence their rRNA genes using newly designed G-AMF- and M-AMF-specific primers. They detected both G-AMF and M-AMF rRNA gene sequences within the same vesicle, suggesting an unexpected co-occurrence. *Colletotrichum tofieldiae* (Ct) is a fungal root endophyte that facilitates phosphorus acquisition in *Arabidopsis thaliana* under conditions of low phosphorus availability (Hiruma et al. 2016). Sarkodee-Addo et al. (2025) examined the impact of Ct inoculation on the growth of komatsuna (*Brassica rapa* var. *perviridis*), as well as on microbial activities. The inoculation with Ct enhanced growth under both phosphorus-deficient and phosphorus-sufficient conditions, possibly by enhancing phosphorus-solubilizing activity in rhizosphere microorganisms. The endospores of *Bacillus pumilus* TUAT1 have been commercially utilized as a biofertilizer. Agake et al. (2025) examined the mechanisms underlying the plant growth-promoting effects of the spores and vegetative cells of TUAT1 by analyzing their growth-promoting effects and the bacterial and fungal communities in soil. The authors found that even the dead cells influence soil microbial communities, suggesting indirect mechanisms of TUAT1 through the modulation of microbial interactions. Farmland manure application is a prevalent agricultural practice that supplies essential nutrients to crops and enhances soil fertility. Okamoto et al. (2025) examined the impact of farmland manure application on the nitrogen fixation of soil microorganisms, revealing that continuous application increased nitrogen fixation capacity through alterations in soil diazotroph communities.

Plant protection from pathogens is one of the well-characterized beneficial activity conferred by beneficial microbes. Takeuchi and Seo (2025) explained the protective rhizosphere bacteria *Psuedomonas protegens*, which produce a wide range of secondary metabolites and extracellular enzymes, especially focusing on how exogenous application of amino acids influences the protective activity. Ahsan et al. (2025) reported dual benefits of *Lysinibacillus xylanilyticus* strain GIC41 in mitigating Pythium root rot and enhancing plant growth in various cultivation methods such as soil pots and hydroponic systems.

Developing new technologies is essential for understanding plant-microbe molecular interactions and harnessing them for agricultural applications. Hairy root transformation is a technique employed in plant biotechnology to produce genetically modified roots with distinct characteristics and widely used for genetic analysis in root-rhizobia symbiosis. Araragi et al. (2025) demonstrated a rapid and efficient method for hairy root transformation applicable to legume crops, including 12 soybean, 1 cowpea, and 1 mungbean cultivar. This was achieved by optimizing conditions such as maintaining adequate humidity at the injection site during cocultivation and adjusting nitrogen levels. Raman spectroscopic analysis offers significant insights into molecular composition and metabolic activities. Suwa et al. (2025) utilized this technique for the non-invasive examination of soybean nodules at the single-cell level. By employing random forest regression, it was demonstrated that biomolecular contributions influence acetylene reduction activity. Additionally, the analysis revealed that polyhydroxybutyrate content fluctuated with plant growth, potentially serving as an indicator of acetylene reduction activity.

Recent studies have highlighted that specific plant-microbe interactions are not fixed to a single stable outcome but rather occur along a continuum from mutualism and commensalism to parasitism. Osayande et al. (2025) summarized this emerging concept, focusing on the mechanisms underlying lifestyle transitions, and discussed how elucidating these mechanisms could contribute to sustainable agriculture and ecosystem management.

Given the limited availability and significant environmental impacts of chemical fertilizers and pesticides, there is an urgent need to develop alternative strategies for sustainable agriculture. We believe that harnessing beneficial microbes offers a promising path forward; however, a detailed molecular-level understanding of how these microbes promote plant growth and health without transitioning to a pathogenic state is essential. We hope that this special issue will advance the field of plant-microbe interactions, in concert with plant biotechnology and other scientific disciplines.
